# Bacterial Contamination of Mobile Phones Used by Healthcare Workers in Critical Care Units: A Cross-Sectional Study from Saudi Arabia

**DOI:** 10.3390/microorganisms11081986

**Published:** 2023-08-02

**Authors:** Nabil Dhayhi, Nader Kameli, Mohammed Salawi, Amjad Shajri, Vinod Kumar Basode, Abdullah Algaissi, Edrous Alamer, Majid Darraj, Khalid Shrwani, Abdulaziz H. Alhazmi

**Affiliations:** 1 Infectious Diseases Unit, Department of Pediatric Medicine, King Fahad Central Hospital, Jazan 45142, Saudi Arabia; 2 Department of Medical Laboratory Technology, Faculty of Applied Medical Sciences, Jazan University, Jazan 45142, Saudi Arabia; 3 Medical Research Center, Jazan University, Jazan 45142, Saudi Arabia; 4 Department of Pediatric Medicine, King Fahad Central Hospital, Jazan 45142, Saudi Arabia; 5 Department of Medicine, Faculty of Medicine, Jazan University, Jazan 45142, Saudi Arabia; 6 Saudi Public Health Authority, Vector-Borne Diseases Laboratory, Jazan 45142, Saudi Arabia; 7 Department of Microbiology and Parasitology, Faculty of Medicine, Jazan University, Jazan 45142, Saudi Arabia

**Keywords:** hospital-associated infections, mobile phones, healthcare workers, bacterial contamination, Jazan, Saudi Arabia

## Abstract

Background: Healthcare-associated infections (HAIs) present a formidable challenge for healthcare institutions, resulting in heightened mortality, morbidity, and economic burden. Within healthcare settings, various equipment and materials, including mobile phones, can potentially act as sources of infection. This study sought to examine the occurrence of bacterial contamination on mobile phones utilized by healthcare workers (HCWs) in intensive care units (ICUs), pediatric intensive care units (PICUs), neonatal intensive care units (NICUs), and cardiac care units (CCUs) within a central hospital (CH) and two peripheral hospitals (PHs) situated in the southwestern province of Saudi Arabia. Materials and methods: We collected a total of 157 samples from mobile phones utilized by HCWs across all ICUs in the CH and PHs. These samples underwent bacteriological analysis to evaluate the degree of bacterial contamination. Results: We found that 45 out of 55 samples from physicians (81.81%) and 58 out of 77 samples from nurses (75.32%) showed bacterial contamination. Contamination rates on HCWs’ mobile phones in the ICU, PICU, and NICU departments of the CH were observed at 69.56%, 80.95%, and 70.27%, respectively. Furthermore, the overall contamination rates in the ICUs, NICUs, and CCUs of the PHs were 78.26%, 88.88%, and 66.66%, respectively. The overall contamination rates of mobile phones in the CH and PHs were 72.11% and 81.13%, respectively. Conclusion: These findings underscore the necessity of routinely disinfecting the mobile phones of HCWs to mitigate the risk of cross-contamination. Implementing robust disinfection protocols can significantly contribute to curtailing the propagation of bacterial pathogens and reducing the incidence of HAIs in healthcare settings.

## 1. Introduction

Healthcare institutes face a significant challenge in healthcare-associated infections (HAIs), which can have considerable consequences regarding mortality, morbidity, and economic cost [1]. HAIs threaten patient safety and can lead to increased healthcare burden. In the healthcare system, mobile phones have become essential tools for communication and have a beneficial impact on patient care. It has been reported that 47.4% of healthcare professionals use their mobile phones more than five times while working in the intensive care unit (ICU) [2]. The convenience and versatility of mobile phones in healthcare settings have made them an integral part of daily clinical practice. The use of mobile phones for HCWs is not limited to communication in modern healthcare settings. However, mobile phones could facilitate quick access to medical information and medical apps that could aid in diagnosis and treatment, enhancing patient care, clinical decision making, and overall workflow efficiency for HCWs and any given health system.

Hand hygiene is one of the most essential preventive interventions against the spread of infections in healthcare settings [3,4]. Proper hand hygiene practices significantly reduce the transmission of pathogens and prevent HAIs. However, in healthcare settings, various medical devices, including stethoscopes and mobile phones, are frequently handled by healthcare providers. Recent studies have highlighted the potential role of mobile phones used by healthcare workers, or even in the community, in transmitting pathogenic bacteria [5,6,7]. The proximity of mobile phones to healthcare providers’ hands and the frequent handling during patient care activities make them susceptible to contamination.

It has been demonstrated that mobile phones used by healthcare workers can serve as reservoirs for nosocomial pathogens [8]. Pathogenic bacteria can easily adhere to the surfaces of mobile phones and be transmitted from the phone of the HCW to the patient or spread between different departments through hand contact [9]. These trends elicit concern in researchers about the exact role of mobile phone contamination in transmitting HAIs within healthcare facilities. Some hospital units, such as critical care, operating rooms, ICUs, and burn units, are particularly vulnerable to HAIs due to patients’ compromised health status and invasive medical devices.

Furthermore, the risk of HAIs may increase in critical care units such as neonatal intensive care units (NICUs), pediatric intensive care units (PICUs), and cardiac care units (CCUs) [10]. These units contain vulnerable populations, including premature newborns, immunocompromised patients, and individuals with other immune system conditions. Despite the importance of mobile phone contamination to transmit pathogens in these critical care areas, there is a lack of comprehensive studies investigating the extent of contamination among HCWs’ mobile phones in our region. In Saudi Arabia, limited studies have explored the bacterial contamination of mobile phones used by HCWs in ICUs, PICUs, NICUs, and CCUs. Some studies have focused on community mobile phone contamination but have not explicitly targeted HCWs’ devices [11]. Other studies have used questionnaire-based approaches to evaluate the role of mobile phones in transmitting infections [12]. Therefore, there is a need for a comprehensive study to examine the bacterial contamination of mobile phones used by healthcare workers in the ICUs, PICUs, NICUs, and CCUs of hospitals in the southwestern province of Saudi Arabia.

Hence, this research aims to fill the knowledge gap regarding mobile phone contamination among HCWs in the region and provide essential data on the prevalence of bacterial contamination in the ICUs, PICUs, NICUs, and CCUs of a central hospital (CH) and two peripheral hospitals (PHs) in the southwestern province of Saudi Arabia. Understanding the extent and nature of contamination will help develop targeted infection control strategies and guidelines to mitigate the risks associated with mobile phone-mediated transmission of healthcare-associated pathogens.

## 2. Materials and Methods

### 2.1. Samples, Data Collection, and Participants’ Inclusion and Exclusion Criteria

After obtaining permission from the hospital administration in the included hospitals, the researchers visited the targeted intensive care wards during the team-round time (between 10 a.m. and 12 p.m.) to recruit volunteers from all levels of HCWs who work in the intensive care wards, including physicians, nurses, respiratory therapist, physiotherapist, and social workers. To maintain confidentiality and avoid pre-swabbing mobile cleaning, we kept the visit’s purpose, date, and time confidential until its commencement. We included HCWs who worked in the units at the visit and excluded non-HCWs or HCWs who refused to participate. We identified HCWs by their badges of direct contact with patients, such as doctors, nurses, respiratory therapists, and physician assistants. A standardized questionnaire was administered to each participant to collect demographic information, including age, gender, and occupation. Information regarding mobile phone cleaning practices, such as frequency and methods, was also collected. We collected swab samples from the mobile phones of the participating healthcare workers using sterile cotton swabs moistened with sterile standard saline solution. The swabs were rubbed over the entire surface of the mobile phone, including the screen, keypad, and back cover. During sample collection, we avoided contact with the participant’s hand. After collection, the swabs were immediately placed in a proper transport media and transported to the laboratory for processing.

### 2.2. Laboratory Procedures

Each swab sample was streaked in the laboratory onto appropriate culture media, including blood agar, MacConkey agar, and chocolate agar (Saudi Prepared Media Laboratory Company, Riyadh, Saudi Arabia). The media were then incubated at 37 °C for 24 to 48 h. After incubation, the colonies were identified on the basis of morphological characteristics, including colony shape, size, color, hemolysis patterns, and Gram staining. Further identification of the isolates was performed using standard biochemical tests, including catalase, coagulase, oxidase, and API identification systems (BioMérieux, Craponne, France). Final identification and antibiotic sensitivity profiles were established using the VITEK^®^2 automated identification system (BioMérieux, Craponne, France) for first identification with appropriate cards for Gram-positive and Gram-negative bacteria according to the manufacturer’s instructions. The Microscan Walkaway automated system (Beckman Coulter, Inc., Sacramento, CA, USA) was used for further confirmation and antimicrobial susceptibility patterns following the manufacturer’s instructions.

### 2.3. Data Analysis

Data were entered into Microsoft Excel and analyzed using SPSS version 23 (IBM Corp., Armonk, NY, USA). Descriptive statistics, including frequencies and percentages, were used to summarize the demographic characteristics of the participants and the prevalence of bacterial contamination on mobile phones. Chi-square or Fisher’s exact tests were used to analyze the association between categorical variables. A *p*-value < 0.05 was considered statistically significant.

### 2.4. Ethical Approval

For this study, we obtained ethical approval from the Jazan Health Ethics Committee in Saudi Arabia (Permission number 077), and our study was conducted according to the Declaration of Helsinki. Before participation, all participants were fully informed about the study’s objectives, ensuring their privacy and confidentiality. They were also allowed to withdraw from the study at any time if they desired. Data collectors did not gather any identifying or personal information from the participants. Only the study investigators could access the shared document where the data were securely stored.

## 3. Results

In Table 1, we summarized the demographic characteristics, work experience, mobile phone cleaning frequency, and professions of the HCWs. Among the participants, 110 (70.06%) were aged less than 35 years, 42 (26.75%) were between 35 and 50 years old, and five (3.18%) were above 50 years old. In terms of work experience, 104 (66.24%) HCWs had less than 10 years of experience, 46 (29.29%) had 10–20 years of experience, and seven (4.45%) had more than 20 years of experience. The cleaning frequency of mobile phones varied, with 15 (9.55%) HCWs reporting daily disinfection, 82 (52.22%) using a dry cloth or tissue daily, 24 (15.28%) cleaning their phones weekly, 19 (12.10%) cleaning them irregularly during the month, and 17 (10.82%) reporting never cleaning or not recalling their cleaning frequency. Among the included HCWs, physicians accounted for 35.03% of the total sample size, while nurses constituted 49.04% of the total individuals. The category labeled “other healthcare workers” encompassed a diverse group of healthcare professionals, as listed in Table 1, and they contributed to 15.92% of the included subjects.

Table 2 presents the bacterial contamination of mobile phones among HCWs. The contamination rate with Gram-negative bacilli was 13.37%, and the overall bacterial contamination rate was 75.15%. Among male HCWs, 34 (80.95%) of 42 samples showed bacterial contamination, while, among female HCWs, 84 (73.04%) of 115 samples were contaminated. Regarding occupation, bacterial contamination was observed in 45 (81.81%) of 55 samples from physicians and 58 (75.32%) of 77 samples from nurses. The contamination rates varied among different departments and hospitals. In the ICUs, mobile phones of HCWs from the CH had a contamination rate of 69.56% (32 of 46 samples), while, in the PH, the rate was 78.26% (18 of 23 samples). Similar trends were observed in the NICUs and CCUs. Overall, mobile phones of HCWs from the CH had a contamination rate of 72.11% (75 of 104 samples), while, in the PH, the rate was 81.13% (43 of 53 samples). There was no significant difference in the prevalence of bacterial contamination among the different ICUs (*p* > 0.05). However, there was a significant association between the duration of mobile phone usage and bacterial contamination (*p* = 0.024).

Gram-positive bacteria were found on 97 (82.19%) mobile phones of HCWs, while Gram-negative bacteria were present on 21 (17.77%) phones, as shown in Table 3. Among the Gram-positive bacteria, *Staphylococcus aureus* was detected in two (1.69%) samples, and coagulase-negative *Staphylococcus* was found in 95 (80.50%) samples. Among the Gram-negative bacteria, *Pseudomonas stutzeri* was the most prevalent, accounting for 17 (14.40%) samples, followed by *E. coli* (two samples, 1.69%), *Klebsiella pneumoniae* (one sample, 0.84%), and *Proteus mirabilis* (one sample, 0.84%).

The antibiotic resistance patterns of Gram-negative and Gram-positive bacterial isolates are presented in Table 4 and Table 5, respectively. Among the Gram-negative bacteria, *Pseudomonas stutzeri* resisted imipenem (10%) and aztreonam (10%). *Proteus mirabilis* strains were resistant to aztreonam (100%), ampicillin (100%), and amoxicillin/clavulanic acid (100%). Among the Gram-positive bacteria, coagulase-negative *Staphylococcus* strains exhibited resistance to multiple antibiotics, including oxacillin (50%), clindamycin (12%), ampicillin (65%), azithromycin (43%), fusidic acid (81%), and erythromycin (58%).

## 4. Discussion

The contamination of mobile phones in healthcare settings has become a growing concern due to their potential role in transmitting HAIs. There has been a scarcity of studies assessing the exact epidemiology of HAIs and the underlying factors contributing to their occurrence in Saudi Arabia. For instance, a study conducted in 2017 by Alshamrani et al. across six different hospitals in Saudi Arabia reported a prevalence rate of 6.8% for HAIs, affecting 114 out of 1666 patients. Among these HAIs, approximately 19.2% were attributed to device-associated infections, highlighting the substantial role of medical equipment in the transmission dynamics of nosocomial pathogens within healthcare settings. Notably, these infections significantly came with a higher prevalence in intensive care departments [13].

In comparison to other regions, the burden of HAIs in Saudi Arabia is comparable to the rates reported in large-scale studies conducted in European countries (6.0%) and the United States (4.0%) [13]. Thus, given the potential impact of HAIs on patient care, healthcare outcomes, and overall healthcare costs, understanding the prevalence and determinants of HAIs is crucial. Investigating the contamination of mobile phones used by HCWs emerges as a vital aspect of this inquiry, as these devices are ubiquitous in healthcare settings and can act as potential reservoirs for pathogenic microbes, including bacteria. Research into the extent of bacterial contamination on HCWs’ mobile phones within specific units, such as intensive care wards, including pediatric, neonatal, and cardiac care wards, can provide valuable insights into the dynamics of HAI transmission [13]. Consequently, several national and international studies have investigated the bacterial contamination of mobile phones, or HCWs’ knowledge and attitudes toward this problem, in various hospital settings, including ICUs, PICUs, NICUs, and CCUs [14,15,16,17,18,19,20,21,22,23,24,25,26,27]. These studies have highlighted the risk of mobile phones serving as reservoirs for nosocomial pathogens, which can be easily transmitted from the phone to patients and spread between different wards through hand contact [9,10]. In the current study, the prevalence of mobile phone bacterial contamination was 75%, higher than what was previously reported in Saudi Arabia. For example, Sadat Ali et al. conducted an older study in 2010, finding a 43% prevalence of mobile phone bacterial contamination [14]. However, Banawas et al. reported a majority of 63% of participants mobiles being contaminated with bacteria in a study conducted in 2018 [19]. The regional and international figures seem similar to what was reported in Saudi Arabia. For instance, in Kuwait, the mobile phone contamination of HCWs in the ICUs was reported at 72.1% [10], while it is said to be 50.45% in Spanish hospitals [17] and 80% in Croatia [18]. Thus, we believe a noticeable increase in bacterial contamination on mobile phones may be attributed to several factors. First, HCWs frequently handle medical devices and accessories such as mobile phones during patient care activities. This frequent handling increases the risk of transferring pathogens from contaminated surfaces to mobile phones. Second, inadequate hand hygiene practices among HCWs can further contribute to the contamination of mobile phones. Poor hand hygiene compliance has been identified as a significant factor in transmitting healthcare-associated pathogens [3,4].

The role of mobile phones as a vector for HAIs Is influenced by various factors, including the type of pathogen, the duration of phone use, and the presence of other infection prevention measures in healthcare settings [21]. This study also aimed to examine factors associated with bacterial contamination of mobile phones used by HCWs. Unfortunately, we were not able to measure the association between the duration of phone use and contamination; however, regarding the nature of the occupation, the current study found that the contamination of Gram-negative bacteria on nurse’ mobile phones was significantly higher (19.48%) than physicians’ mobile phones (7.69%). However, when considering the overall bacterial contamination (including both Gram-positive and Gram-negative bacteria), the mobile phones of nurses (75.32%) had a slightly lower contamination rate than those of physicians (81.81%). In line with these findings, a previous study conducted in Saudi Arabia also reported somewhat lower overall bacterial contamination on nurses’ mobile phones (41.80%) compared to the mobile phones of physicians (51.3%) [15], and this is consistent with another study in the same region [19]. This finding could be explained by the nature of nurses’ work when they often spend more time in direct patient care activities, such as bedside care, administering medications, and performing procedures.

Regarding the association of the hospital setting and wards with mobile phone contamination, the prevalence of overall bacterial contamination on mobile phones used by HCWs in the NICU of CH was 70.27%, including 13.04% of Gram-negative bacterial contamination. In contrast, the prevalence of overall bacterial contamination on mobile phones used by HCWs in NICU in PHs was 88.88%, including 11.11% Gram-negative bacterial contamination. Similarly, a study conducted in Kuwait hospitals reported an overall bacterial contamination rate of 79.6% on mobile phones used by HCWs in the NICU [10]. These differences depending on the location and hospital settings were also observed in a study conducted in the central region of Saudi Arabia, where the prevalence of contamination of bacteria on mobile phones of HCWs in three different hospitals was 78.23%, 70%, and 35.39% [19]. Another factor that may affect HCWs’ mobile phone contamination is their habit of cleaning their mobile phones. We found that 52.22% of HCWs cleaned their phones daily with dry cloth or tissue, while 15.28% and 12.10% cleaned their phones weekly or at irregular times per month. Kotris et al. reported that 33.33% of medical students use dry clothes to clean their mobile phones [18]. The present study supports previous reports that the majority of HCWs do not use disinfectant to clean their mobile phones regularly [2,22], and 53% of HCWs never cleaned their mobile phones reported in a previous study [23] compared to 10.82% of HCWs who said that they never cleaned their mobile phones. It is important to note that using 70% isopropyl alcohol can be effective as a disinfectant. In previous studies, no microorganisms were detected when mobile phones were cleaned with 70% alcohol [1,25].

The current study identified six different human pathogens and others considered normal flora on the mobile phones of healthcare personnel. The prevalence of Gram-positive and Gram-negative bacteria was 82.19% and 17.77%, respectively. These results contrast another study conducted in the central region of Saudi Arabia, which reported a prevalence of 89.47% for Gram-positive bacteria and 10.53% for Gram-negative bacteria [19]. Banawas et al. found coagulase-negative staphylococci (60.50%), *Staphylococcus aureus* (2.6%), and other Gram-positive (26.4%) and Gram-negative (7%) bacteria on mobile phones [19]. The current study detected *Staphylococcus aureus* (1.69%), coagulase-negative *Staphylococcus* (80.50%), *Pseudomonas stutzeri* (14.40%), *E. coli* (1.69%), *Klebsiella pneumoniae* (0.84%), and *Proteus mirabilis* (0.84%) on the mobile phones. These findings are consistent with a previous report that identified coagulase-negative *Staphylococcus* as the most common isolates from contaminated mobile phones of medical students in Saudi Arabia, as well as other reports where *Staphylococcus aureus* and coagulase-negative *Staphylococcus* have commonly detected bacteria on mobile phones [20,21]. Although some microorganisms isolated in this study are known to be infectious agents and capable of causing nosocomial infections and severe complications, especially in immunocompromised individuals, none of the bacterial isolates were highly resistant to antibiotics, and none were identified as Gram-negative extended-spectrum beta-lactamase (ESBL)- or methicillin-resistant *Staphylococcus aureus* (MRSA). Thus, further research is needed to routinely investigate pathogenic bacteria that can be found on healthcare workers’ mobile phones in hospital settings.

It is noteworthy that incorporating antimicrobial stewardship and infection control programs in healthcare facilities is paramount in safeguarding public health and combating the rising threat of antimicrobial resistance and HAI [28,29,30]. These programs play a vital role in optimizing antimicrobial use, prioritizing patient safety, and preserving the effectiveness of existing antibiotics. Antimicrobial stewardship initiatives focus on a multidisciplinary approach to ensure appropriate prescribing practices, reduce unnecessary antimicrobial exposure, and minimize the emergence of resistant pathogens. Moreover, infection control programs complement these efforts by implementing measures to avoid and limit the spread of infections within healthcare settings. The Italian experience, as described by Albano et al. in their study conducted between 2020 and 2021, highlights the potential for fruitful outcomes from effective antimicrobial stewardship programs, leading to improvements in clinical, microbiological, and economic measures. Strong adherence to the antimicrobial stewardship program recommendations is associated with better outcomes. Furthermore, recommendations suggest that the design of antimicrobial stewardship programs should be based on the local status of antimicrobial use and resistance [28]. Successful outcomes of such programs necessitate frequent interactions among multidisciplinary teams involving infectious diseases specialists, clinical pharmacists, clinical microbiologists, and infection control practitioners under the governance of hospital administration. According to a systematic review (included studies between 2000 to 2014) conducted by Nathwani et al. assessing the economic and clinical impact of antimicrobial stewardship programs, only 14% of these studies originated from Asia, with two came from Saudi Arabia and none in the southwestern region of Saudi Arabia. The review concluded that hospital antimicrobial stewardship programs have positively impacted clinical and economic outcomes [30,31,32].

Despite the significant findings of our study, several limitations need to be acknowledged. First, our study focused on bacterial contamination of mobile phones and did not assess the presence of other types of pathogens, such as viruses or fungi or even other uncultivable bacteria. Future studies could explore the full spectrum of microorganisms on mobile phones in healthcare settings and should include newly investigated uncultivable bacterial species associated with chronic infections [33]. Second, our study was conducted in a specific geographical region and may not represent the entire country or other healthcare settings globally. The prevalence and types of bacterial contamination on mobile phones may vary in regions and healthcare facilities. Therefore, caution should be exercised when generalizing our findings to other contexts. Third, we only assessed the bacterial contamination of mobile phones and did not investigate the transmission of infections from mobile phones to patients. While previous studies have suggested a potential link between mobile phone contamination and HAI transmission [34], further research is needed to establish a definitive causal relationship.

Longitudinal studies should be conducted and could be crucial in comprehending the dynamic nature of mobile phone contamination trends. Conducting such studies will aid in monitoring changes in contamination rates, identifying potential seasonal variations, and assessing the effectiveness of various interventions to reduce contamination. Through such action, healthcare institutions can tailor their infection control strategies accordingly and ensure sustained improvements in mobile phone hygiene practices. Future research should include and analyze the impact of unculturable bacterial species and viral and fungal pathogens to better understand mobile phone contamination in healthcare settings and intensive care wards. Utilizing phenotypic and genotypic identification techniques can help identify a broader range of microorganisms on mobile phones, including potential pathogenic viruses or highly transmissible resistant bacteria. Understanding the diverse array of pathogens present on mobile phones will be critical in devising targeted and effective disinfection protocols. While investigating the presence of pathogens on mobile phones is essential, understanding the transmission dynamics from contaminated phones to patients or healthcare workers is equally critical. This information can illuminate the potential transmission routes and identify high-risk activities or patient care scenarios where we should strengthen infection control measures. Moreover, studying transmission dynamics will aid in determining the role of mobile phones in propagating healthcare-associated infections, which, in turn, can inform infection prevention policies. Educational interventions can play a pivotal role in improving mobile phone hygiene practices among healthcare personnel. Designing and implementing targeted educational programs to raise awareness about the importance of regular mobile phone disinfection can empower HCWs to take proactive measures. Evaluating the effectiveness of these interventions through follow-up assessments and feedback mechanisms will enable healthcare institutions to refine their educational approaches and ensure sustained behavioral changes among HCWs.

## 5. Conclusions

In conclusion, our study sheds light on the alarming extent of bacterial contamination on mobile phones used by HCWs in critical care wards in Saudi Arabia. These findings underscore the vital need for robust infection control measures within healthcare settings, with particular attention to the regular cleaning and disinfection of mobile phones. By implementing effective strategies to minimize the risk of HAIs, we can safeguard patients and HCWs from potential harm. It is crucial to prioritize infection control measures, including regular cleaning and disinfection of mobile phones. Further research is needed to understand the impact of mobile phone contamination on HAI transmission and to develop effective strategies for maintaining mobile phone hygiene in healthcare settings. Further research should investigate viral and fungal contamination on mobile phones and explore transmission dynamics.

## Figures and Tables

**Table 1:** Demographic characteristics, work experience, mobile phone cleaning frequency, and the profession of healthcare workers.

Variables	Number (%)
Age	
Less than 35 years	110 (70.06)
35–50 years	42 (26.75)
More than 50 years	5 (3.18)
Work Experience	
Less than 10 years	104 (66.24)
10–20 years	46 (29.29)
More than 20 years	7 (4.45)
Cleaning frequency	
Daily with disinfectant	15 (9.55)
Daily with dry cloth or tissue	82 (52.22)
Weekly with dry cloth or tissue	24 (15.28)
Irregular times per month, either dry cloth or tissue	19 (12.10)
Never or I do not remember	17 (10.82)
Occupation	
Physicians	55 (35.03)
Nurses	77 (49.04)
Other healthcare workers ^#^	25 (15.92)

^#^ Respiratory therapists, physiotherapists, occupational therapists, and social workers.

**Table 2:** Bacterial contamination of mobile phones of healthcare workers.

Characteristic	Contamination of Mobile Phones of Healthcare Workers with Gram-Negative Bacilli *	Overall Bacterial Contamination of Mobile Phones of Healthcare Workers *	*p*-Value
Gender			
Male	6 (14.28)	34 (80.95)	0.15654
Female	15 (13.04)	84 (73.04)	
Occupation			
Physicians	4 (7.69) ^a^	45 (81.81)	0.1891
Nurses	15 (19.48) ^a^	58 (75.32)	
Other healthcare workers	2 (8.00)	15 (60)	
Central hospital			
ICU	6 (13.04)	32 (69.56)	0.6588
PICU	3 (14.28)	17 (80.95)	
NICU	4 (10.81)	26 (70.27)	
Peripheral hospitals			
ICU	3 (13.00)	18 (78.26)	0.8344
NICU	1 (11.11)	8 (88.88)	
CCU	4 (19.04)	17 (80.95)	
Hospitals			
Central hospital	13 (12.50)	75 (72.11)	0.1094
Peripheral hospitals	8 (15.09)	43 (81.13)	
Total	21 (13.37)	118 (75.15)	

* Percentages are given in parentheses; ^a^ significant (*p* = 0.0246).

**Table 3:** Gram-positive and Gram-negative bacterial isolates from the mobile phones of healthcare workers.

Name of the Bacteria	Number (%)
Gram-positive bacteria	97 (82.19)
*Staphylococcus aureus*	2 (1.69)
Coagulase-negative *Staphylococcus*	95 (80.50)
Gram-negative bacteria	21 (17.77)
*Pseudomonas stutzeri*	17 (14.40)
*E. coli*	2 (1.69)
*Klebsiella pneumoniae*	1 (0.84)
*Proteus mirabilis*	1 (0.84)

**Table 4:** Antibiotic resistance of Gram-negative bacterial isolates from mobile phones of healthcare workers.

Antibiotic	Dose (µg/disc)	*Pseudomonas stutzeri* (n = 17) % Resistance	*E. coli* (n = 2) % Resistance	*Klebsiella pneumoniae* (n = 1) % Resistance	*Proteus mirabilis* (n = 1) % Resistance
Ceftazidime	10	0	0	0	0
Cefepime	50	0	0	0	0
Amikacin	10	0	0	0	0
Gentamicin	50	0	0	0	NA
Tigecycline	15	0	0	0	0
Meropenem	10	0	0	0	0
Ciprofloxacin	5	0	0	0	NA
Piperacillin/tazobactam	100/10	0	0	0	NA
Imipenem	10	10	0	0	0
Aztreonam	30	10	0	0	100
Ampicillin	10	NA	0	0	100
Amoxicillin/clavulanic acid	20–10	NA	0	0	100

**Table 5:** Antibiotic resistance of Gram-positive bacterial isolates from mobile phones of healthcare workers.

Name of the Antibiotic	Dose (Microgram/Disc)	*Staphylococcus aureus* (n = 2) % Resistance	*Coagulase-negative Staphylococcus* (n = 95) % Resistance
Oxacillin	1	0	50
Clindamycin	2	0	12
Linezolid	30	0	0
Vancomycin	30	0	0
Rifampicin	5	0	0
Ampicillin	10	0	65
Azithromycin	15	0	43
Fusidic acid	10	0	81
Erythromycin	15	0	58

## Data Availability

Data are available upon reasonable request from the authors.
